# Mitochondrial activity regulates the differentiation of skin-derived mesenchymal stem cells into brown adipocytes to contribute to hypertension

**DOI:** 10.1186/s13287-021-02169-0

**Published:** 2021-03-10

**Authors:** Wenda Xi, Wendong Chen, Weihong Sun, Xiangxiao Li, Zhimin Suo, Gonghao Jiang, Pingjin Gao, Qun Li

**Affiliations:** 1 The Department of Cardiovascular Medicine, State Key Laboratory of Medical Genomics, Shanghai Key Laboratory of Hypertension, Ruijin Hospital, Shanghai Institute of Hypertension, Shanghai Jiao Tong University School of Medicine, No. 197, Ruijin 2nd Road, Shanghai, 200025 China; 2 Department of Digestion, Huaihe Hospital of Henan University, Kaifeng, 475000 China

**Keywords:** Skin-derived mesenchymal stem cell, Differentiation, Brown adipocyte, Mitochondrial activity, Hypertension

## Abstract

**Background:**

Brown adipocytes (BAs) are major components of brown adipose tissue (BAT), which is involved in blood pressure regulation. BAs are derived from multiple progenitors, including PDGFRα^+^ adipose-derived stem cells (ASCs). Skin-derived mesenchymal stem cells (S-MSCs) have the capacity to differentiate into adipocytes; however, their ability to differentiate into BAs remains unexplored. We aim to study the ability and regulatory mechanism of the differentiation of S-MSCs into BAs and the direct role of BAT in blood pressure regulation.

**Methods:**

Protein expression was measured by flow cytometry or Western blotting, and gene mRNA levels were quantified by real-time quantitative PCR (RT-PCR). To induce the differentiation of S-MSCs into BAs, S-MSCs were stimulated with a brown adipogenic cocktail comprising insulin, IBMX, dexamethasone, triiodothyronine (T3), and rosiglitazone for the indicated periods. The oxygen consumption rate (OCR) was measured with an XF24 Extracellular Flux Analyzer. Mitochondrial mass was determined by flow cytometry and fluorescence staining. Hypertension was induced in WT mice by infusion of angiotensin II (Ang II), and systolic blood pressure (SBP) was measured using a tail cuff. Interscapular brown adipose tissue (iBAT)-deficient mice were generated by surgical removal of the iBAT depot, after which the animals were allowed to recover for 6 days. Aortic, iBAT, and heart tissue sections were analyzed by hematoxylin and eosin (HE) staining.

**Results:**

We found that in vitro, S-MSCs isolated from the mouse dermis expressed the stem cell markers CD90/105 and PDGFRα and readily differentiated into BAs. Mitochondrial biogenesis and oxygen consumption were markedly increased during differentiation of S-MSCs into BAs*.* In vivo, iBAT was converted to white adipose tissue (WAT) in Ang II-induced hypertensive mice. We assessed the direct role of BAT in blood pressure (BP) regulation by using iBAT-deficient mice (generated by surgical removal of iBAT) and C57BL/6 (wild-type (WT)) mice and found that Ang II-induced BP elevation and vascular damage were markedly aggravated in iBAT-deficient mice compared with WT mice.

**Conclusions:**

This study demonstrates that PDGFRα^+^ S-MSCs are able to differentiate into BAs and that this differentiation is regulated by mitochondrial activity. We also show that BAT plays a direct role in ameliorating Ang II-induced hypertension. The therapeutic potential of BAT for the prevention of hypertension-induced organ remodeling thus warrants further investigation.

**Graphical abstract. Schematic of the in vitro differentiation of PDGFRα^+^ S-MSCs into BAs via a process regulated by mitochondrial activity. BAT plays a direct role in Ang II-induced hypertension and target organ remodeling:**

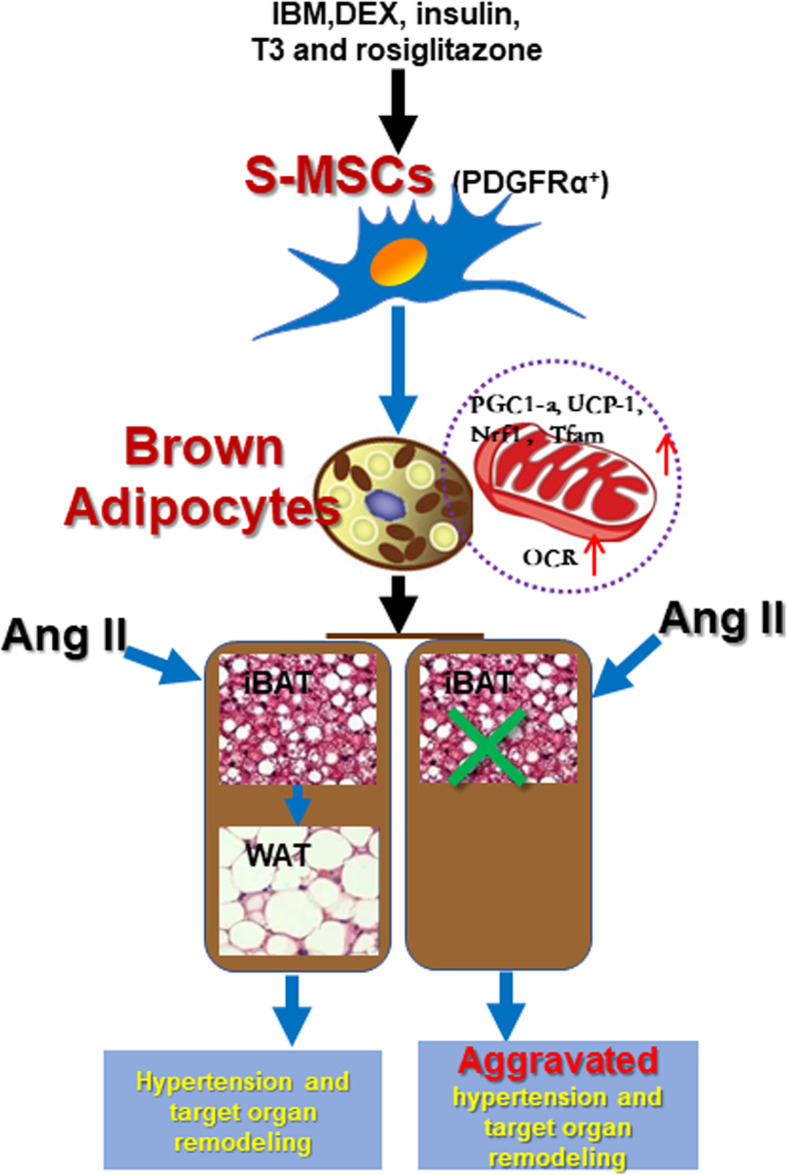

## Highlights

- PDGFRα^+^ S-MSCs are BA progenitors.
- The process by which S-MSCs differentiate into BAs is regulated by mitochondrial activity.
- Brown adipose tissue deficiency aggravates Ang II-induced hypertension and target organ remodeling.

## Background

The adipose organ is composed of WAT and BAT. WAT serves as an energy store for the body, whereas BAT utilizes chemical energy through uncoupled respiration and thermogenesis in all mammals [1]. BAT plays a critical role in energy homeostasis and body weight control via fat thermogenesis and is highly metabolically active, raising the possibility that it may serve as a potential therapeutic target for metabolic diseases [2]. Transgenic ablation of BAT is associated with not only obesity but also systemic hypertension and cardiac fibrosis, as shown in transgenic mice with reduced brown fat [3, 4]. Fibroblast growth factor 21 (FGF21) derived from BAT plays an endocrine-mediated protective role against hypertensive cardiac remodeling in mice [5]. BAs are the major components of BAT and arise from distinct developmental origins, including adipogenic progenitors, myogenic factor 5 (Myf5)^+^ progenitors and neuronal cells [6, 7]. However, the underlying origin of BAs is not completely understood.

Mesenchymal stem cells (MSCs) can be expanded and differentiated into a variety of mesenchymal cell types, such as adipocytes, osteoblasts, and chondrocytes [8]. Human foetal mesenchymal stem cells (fMSCs) have been shown to differentiate into BAs [9]. Previous studies have demonstrated that PDGFRα^+^ ASCs isolated from WAT can differentiate into BAs in vitro and in vivo [10]*.* S-MSCs isolated from the largest lymphoid organ, the skin, share functional similarities with MSCs and also consistently differentiate into adipocytes, osteocytes, and chondrocytes [11, 12]. S-MSCs have the ability to migrate to inflamed tissues and exert immunosuppressive effects to inhibit the development of atherosclerosis, EAE, and hypertension in mice, which indicates that S-MSCs are promising cell sources for stem cell-based therapies for chronic inflammatory diseases and possibly transplantation [12–15]. However, it is unknown whether S-MSCs can differentiate into BAs. Elucidating the specific mechanisms that regulate the differentiation of S-MSCs into BAs is very important for the development of feasible clinical treatments.

According to recent studies, mitochondrial regulation is being increasingly recognized as an important determining factor in stem cell biology and function. There is mounting evidence that mitochondrial metabolism is implicated in the adipogenic differentiation of MSCs [16, 17]. In addition, the differentiation and homeostatic function of adipose tissue are supported by mitochondrial biogenesis [18]. It is also worthwhile to note that BAs are rich in mitochondria, and BAT has been known to play important roles in lipid metabolism and energy expenditure, showing rich expression of thermogenic markers that participate significantly in mitochondrial biogenesis [19, 20]. Studies have demonstrated that mitochondria play an important regulatory role in determining the differentiation capacity of MSCs [17].

Skin is an easily accessible and ideal source of tissue for the isolation of S-MSCs. Thus far, whether S-MSCs located in the dermis can differentiate into BAs and the effect of mitochondrial biogenesis and activity on the differentiation of S-MSCs into BAs have not been reported. In our study, we demonstrate that S-MSCs readily differentiate into BAs, that this differentiation is regulated by mitochondrial activity, and that iBAT directly contributes to improving blood pressure in Ang II-induced hypertensive mice.

## Methods

### Mice

C57BL/6 J (WT) mice (8–12 weeks of age) were purchased from Shanghai SLAC Laboratory Animal Co. (Shanghai, China). All mice were kept under specific pathogen-free (SPF) conditions in compliance with the National Institutes of Health Guide for the Care and Use of Laboratory Animals under the approval (SYXK-2003-0026) of the Scientific Investigation Board of Shanghai Jiao Tong University School of Medicine, Shanghai, China. iBAT-deficient (-BAT) mice were generated by surgical removal of the iBAT depot from C57BL/6 J mice, after which the animals were allowed to recover for 6 days.

### Differentiation of S-MSCs into brown adipocytes

Preparation, culture, and characterization of S-MSCs were performed as described previously [12]. For in vitro differentiation into BAs, S-MSCs were plated in complete medium at a density of 2 × 10^4^ cells per square centimeter in 6-well culture plates and refed every 2 days until they were 100% confluent or postconfluent. BA differentiation was induced by treating cells for 48 h in medium consisting of 10% FBS, 0.5 mM isobutylmethylxanthine (IBM), 1 μM dexamethasone (DEX), 240 IU/μM insulin, 1 nM T3, and 1 μM rosiglitazone (Alexis Biochemicals). After 48 h, the medium was exchanged with medium comprising 10% FBS (Gibco, #10099-141, Australia), 240 IU/μM insulin, 1 nM T3, and 1 μM rosiglitazone for another 6–8 days, with the differentiation medium being replaced every 2 days. Images of cells were obtained with a light microscope and a Zeiss AxioCam MRm camera (Zeiss, Germany).

### Oxygen consumption

S-MSCs were seeded at a density of 4 × 10^4^ cells per well in XF 96-well plates. After induction of differentiation into BAs, the OCR was measured with a Seahorse XF96 Extracellular Flux Analyzer (Seahorse Bioscience, Billerica, MA, USA) following the manufacturer’s instructions. Oligomycin and rotenone were used as inhibitors, and the uncoupler FCCP was used as an agonist. The results were calculated and are presented as basal respiration, ATP production, proton leakage, maximal respiration, and spare capacity.

### Flow cytometry

Flow cytometry was carried out to assess mitochondrial mass using MitoTracker Green FM (MTGFM), a fluorescent mitochondrion-selective probe (Invitrogen, #M7514, USA). S-MSCs were seeded at a density of 4 × 10^5^ cells per well in 12-well plates. After they differentiated into BAs, the cells were suspended in 0.125% trypsin, centrifuged, washed with PBS twice, and stained with 100 nM MTGFM at 37 °C for 30 min. Then, the cells were washed with PBS and stained with propidium iodide at room temperature for 15 min to label the dead cells. The intensity of fluorescence was measured on a Beckman Coulter CytoFLEX instrument, and the mean fluorescence intensity (MFI) was analyzed using FlowJo 10.2 software.

The following antibodies were used to characterize S-MSCs by flow cytometry: FITC-conjugated rat anti-mouse CD105 (MJ718, Abcam, #ab184667), APC-conjugated rat anti-mouse CD90 (R&D, # FAB7335A), APC-conjugated rat anti-mouse CD45 (Biolegend, #103112), and PE-conjugated rat anti-mouse CD140a (PDGFRα, Biolegend, #135906). APC-conjugated goat anti-rat IgG2b, FITC-conjugated goat anti-rat IgG1, and PE-conjugated goat anti-rat IgG1 (all from BD Bioscience) were used as isotype controls.

### RNA extraction and real-time RT-PCR

S-MSCs were plated at a density of 4 × 10^5^ cells per well in 12-well plates for total RNA extraction. Total RNA was extracted with TRIzol reagent (Gibco Life Technologies) following the manufacturer’s instructions. Total RNA (2 μg) was reverse transcribed (RT) into cDNA, and 0.5 μl RT product was used for real-time RT-PCR to measure the mRNA level of each gene. Real-time RT-PCR was performed on a StepOnePlus Real-Time PCR System (Applied Biosystems, USA) using SYBR Green PCR Master Mix (TaKaRa). β-Actin was used as internal control. The expression levels of all genes were normalized to the expression level of the housekeeping gene β-actin. Cycle threshold (CT) was used for data analysis. All cytokine primers were ordered from Invitrogen (Shanghai). The primer sequences were as follows: PPARG coactivator 1 alpha (PGC-1α): sense, 5′-CCCTGCCATTGTTAAGACC-3′, and antisense, 5′-TGCTGCTGTTCCTGTTTTC-3′; uncoupling protein 1 (UCP-1): sense, 5′-ACTGCCACACCTCCAGTCATT-3′, and antisense: 5′-CTTTGCCTCACTCAGGATTGG-3′; nuclear respiratory factor 1 (NRF1): sense, 5′-GCCGTCGGAGCACTTACT-3′, and antisense: 5′-CTGTTCCAATGTCACCACC-3′; transcription factor A (TFAM): sense, 5′-CGCAGCACCTTTGGAGAA-3′, and antisense, 5′-CCCGACCTGTGGAATACTT-3′.

### Immunoblotting

S-MSCs were plated at a density of 8 × 10^5^ cells per well in 6-well plates. After they were differentiated into BAs, the cells were lysed in cell lysis buffer (10 mmol/L Tris-HCl (pH 7.5) 150 mmol/L NaCl, 0.1% SDS, 1% Triton X-100, 2 μg/ml aprotinin, 2 μg/ml leupeptin, and 1 mmol/L PMSF) (Beyotime, Shanghai, China). Protein concentrations were determined using the bicinchoninic acid protein assay (Thermo Fisher Scientific). Aliquots containing 10 μg of total protein were subjected to SDS-PAGE on 12% gels for Western blot (WB) analysis with the following antibodies: anti-PGC1-α (Abcam, #ab54481, 1:1000 dilution), anti-UCP-1 (Abcam, #ab10983, 1:1000 dilution), anti-mitochondrial complex-1 (Proteintech, #15181-1-AP, 1:4000 dilution), anti-mitochondrial complex-4 (Proteintech, #26003-1-AP, 1:2500 dilution), and anti-GAPDH (Proteintech, HRP-60004, 1:4000 dilution). Ang II-stimulated brown adipocytes were lysed in cell lysis buffer and collected for WB analysis with anti-PGC1-α, anti-UCP-1, and anti-GAPDH antibodies. All PVDF membranes (Millipore Sigma) were incubated with horseradish peroxidase-conjugated goat anti-mouse IgG (Proteintech, #SA00001-1, 1:5000 dilution) and goat anti-rabbit IgG (Proteintech, #SA00001-2, 1:5000 dilution) secondary antibodies for 2 h. The immunoreactive bands were detected using an enhanced chemiluminescence detection kit (Perkin Elmer).

### Hypertension induction and blood pressure measurement

Minipumps (1004, Alzet, Cupertino, California) were implanted subcutaneously into mice to deliver Ang II (Sigma) or 0.9% NaCl. Blood pressure (BP) was measured by a tail cuff using the BP-2000 Blood Pressure Analysis System (Visitech Systems, Napa Place Apex, North Carolina). At 10 weeks of age, 12 male C57BL/6 J mice were randomly divided into 2 groups: the WT-C group (control group, infused with 0.9% NaCl, *n* = 6) and the WT-A group (Ang II-infused group, infused with Ang II for 28 days at a rate of 750 ng/kg per minute, *n* = 6). Infusions were administered to investigate the conversion of interscapular brown adipose tissue (iBAT) to white adipose tissue (WAT) in Ang II-induced hypertensive mice. At 10 weeks of age, 24 male C57BL/6 mice were randomly divided into 4 groups: the WT-C group (control group, infused with 0.9% NaCl), the -BAT-34-C group (iBAT-deficient mice that were allowed to recover for 6 days and immediately infused with 0.9% NaCl for another 28 days), the WT-Ang II group (Ang II-infused group, infused with Ang II for 28 days at a rate of 750 ng/kg per minute), and the -BAT-34-Ang II group (iBAT-deficient mice that were allowed to recover for 6 days and immediately infused with Ang II for 28 days at a rate of 750 ng/kg per minute). Noninvasive tail cuff monitoring of the systolic blood pressure (SBP) of the above 4 groups of mice (*n* = 6) was performed.

### Histological analysis

Aortic, iBAT, and heart tissues were fixed with paraformaldehyde and embedded in paraffin, and 6-μm sections were stained with hematoxylin and eosin (HE) [13]. Images of the tissues were obtained by using an Axio Imager 2 upright microscope (Zeiss, Germany) and captured by using an Axiocam 506 color camera (Zeiss, Germany). Images were acquired with ZEN Imaging software (Zeiss, Germany). For mitochondrial staining, S-MSCs were plated at a density of 2 × 10^4^ cells per well in 48-well plates. The mitochondria of undifferentiated S-MSCs and S-MSCs differentiated for 4 days were stained with 200 nM MitoTracker Red CMXRox (Invitrogen, #M7512) at 37 °C for 30 min. The nuclei of the cells were stained with DAPI. For immunofluorescence staining, the cultured S-MSCs were stained with a PDGFRα primary antibody (Abcam, #ab203491) and a polyclonal rabbit anti-goat IgG - H&L secondary antibody (Alexa Fluor® 594) (Abcam, #ab150144). Fluorescence images were captured with an Axio invert microscope and an AxioCam MRm camera (Zeiss, Germany).

### Statistical analysis

The data were analyzed with GraphPad Prism 8 and are presented as the mean ± SEM. Student’s *t* test was used when two conditions were compared, and analysis of variance (ANOVA) with Bonferroni or Newman-Keuls correction was used for multiple comparisons. All data that were analyzed by Student’s *t* test or one-way or two-way ANOVA passed the normality and logarithmic tests (the Shapiro-Wilk test), which were performed by using GraphPad Prism 8. Probability values of < 0.05 were considered significant.

## Results

### PDGFRα^+^ S-MSCs readily differentiated into brown-type adipocytes in vitro

**Fig. 1:**
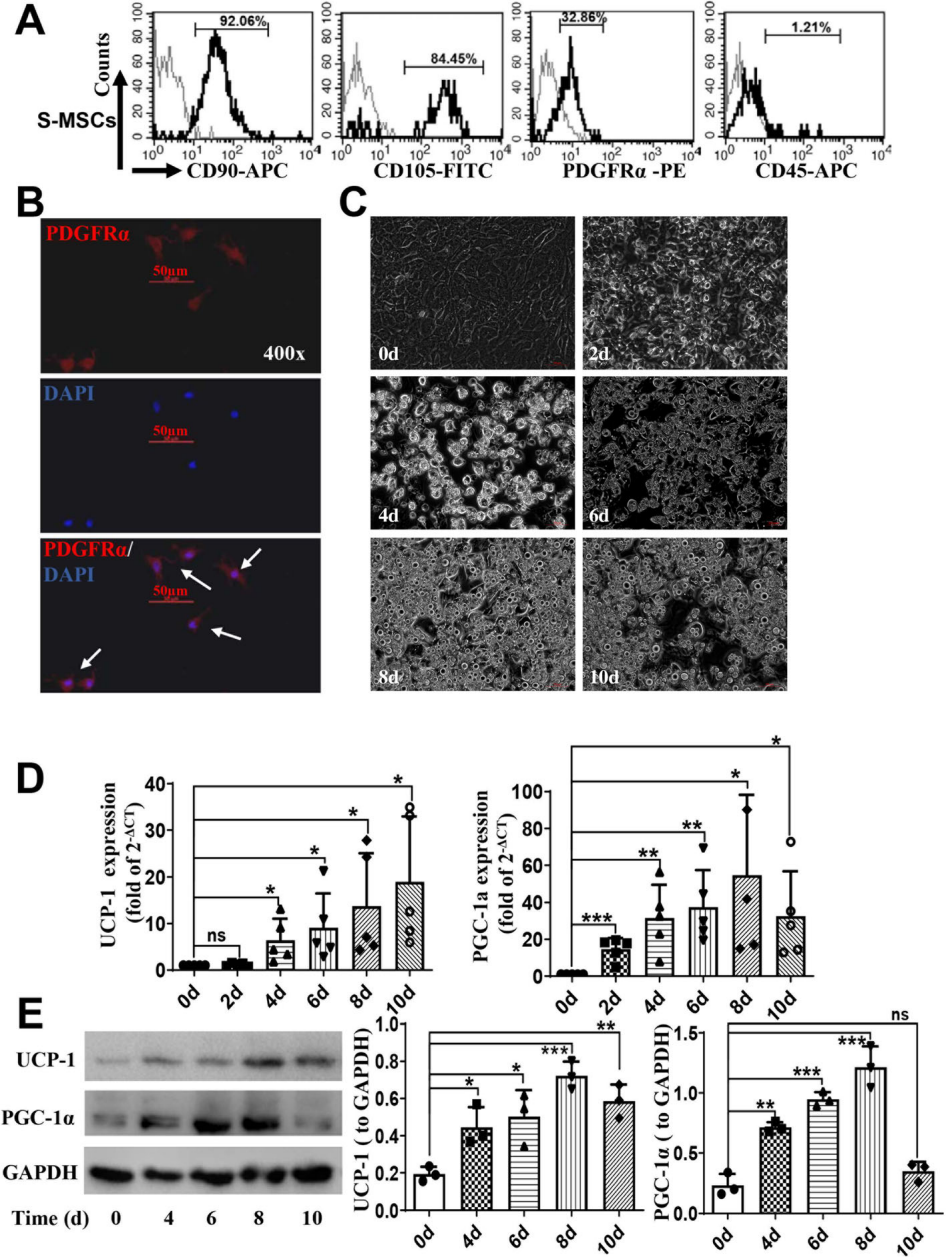
Differentiation of PDGFRα^+^ S-MSCs into brown adipocytes (BAs) in vitro*.* S-MSCs were isolated from the dermis of neonatal mice. **a** The phenotypic characteristics of S-MSCs were assessed by flow cytometry with antibodies against CD90-PE, CD105-PE, PDGFRα-PE, and CD45-PE and corresponding negative IgG control antibodies, which are indicated by gray curves. The *X*-axis indicates the fluorescence intensity (antibody staining). **b** PDGFRα expression was measured by immunofluorescence staining. S-MSCs were stained with PDGFRα and DAPI and visualized by fluorescence microscopy. The bottom row shows merged images of PDGFRα (red) and DAPI (blue, nuclei); the arrows indicate PDGFRα^+^ S-MSCs. Original magnification = × 400; bars = 50 μm. S-MSCs were induced to differentiate for 2, 4, 6, 8, or 10 days with an adipogenic cocktail comprising insulin, IBMX, dexamethasone, triiodothyronine (T3), and rosiglitazone. **c** Images of cells were obtained 0, 2, 4, 6, 8, and 10 days after stimulation by light microscopy. Original magnification = × 100 or × 400. The mRNA levels (**d**) and protein expression (**e**) of PGC-1α and UCP-1 after stimulation of S-MSCs with the adipogenic cocktail were measured by real-time polymerase chain reaction (RT-PCR) and Western blot (WB) analysis, respectively. Densitometric scans of three WBs are quantified in the bar graphs. The results shown are representative of at least three independent experiments. Data that passed the normality test were analyzed by one-way ANOVA (**d**, **e**). *P < 0.05, **P < 0.01, ***P < 0.001; ns indicates not significant

BAs are known to be derived from multipotent progenitor cells, such as PDGFRα^+^ ASCs [10]. S-MSCs are easily attainable MSCs that have been favored recently for stem cell research and the development of tissue therapies [12–15]. We reasoned that S-MSCs can also be induced to differentiate into BAs. Here, we demonstrated by FACS analysis that S-MSCs isolated from the dermis of the mouse skin were positive for the characteristic cell surface markers CD90 (90.06%), CD105 (84.45%), and PDGFRα (32.68%) and negative for CD45 (1.21%). The *X*-axis indicates the fluorescence intensity (antibody staining). The percentage of S-MSCs that were positive for PDGFRα was approximately 32.86%, which means that a substantial portion of the S-MSCs seemed to express PDGFRα at low levels (Fig. 1a). The expression of PDGFRα on S-MSCs was further confirmed by immunofluorescence staining (Fig. 1b). To induce differentiation into BAs, S-MSCs were stimulated with an adipogenic cocktail comprising insulin, IBMX, dexamethasone, triiodothyronine (T3), and rosiglitazone for the indicated periods. Lipid droplets became discernible in the cells after 2 days of stimulation and could be observed in approximately 90% of the cells by day 8, indicating the differentiation of the cells into BAs (Fig. 1c). The thermogenic markers mitochondrial UCP-1 and PGC-1α are considered hallmarks of BAs [9]. We measured the expression of UCP-1 and PGC-1α in the cells during the course of induced differentiation by RT-PCR and found that the levels of UCP-1 and PGC-1α transcripts steadily increased during the 10-day period of differentiation, with a slight drop in PGC-1α levels on day 10 (Fig. 1d). This expression pattern of the mRNAs was largely correlated with the pattern of protein levels during differentiation, with both markers showing a continual increase in levels with a minor drop on day 10 (Fig. 1e). These results showed that S-MSCs express PDGFRα and readily differentiate into BAs upon adipogenic induction in vitro.

### Differentiation of S-MSCs into BAs is accompanied by enhanced oxygen consumption

**Fig. 2:**
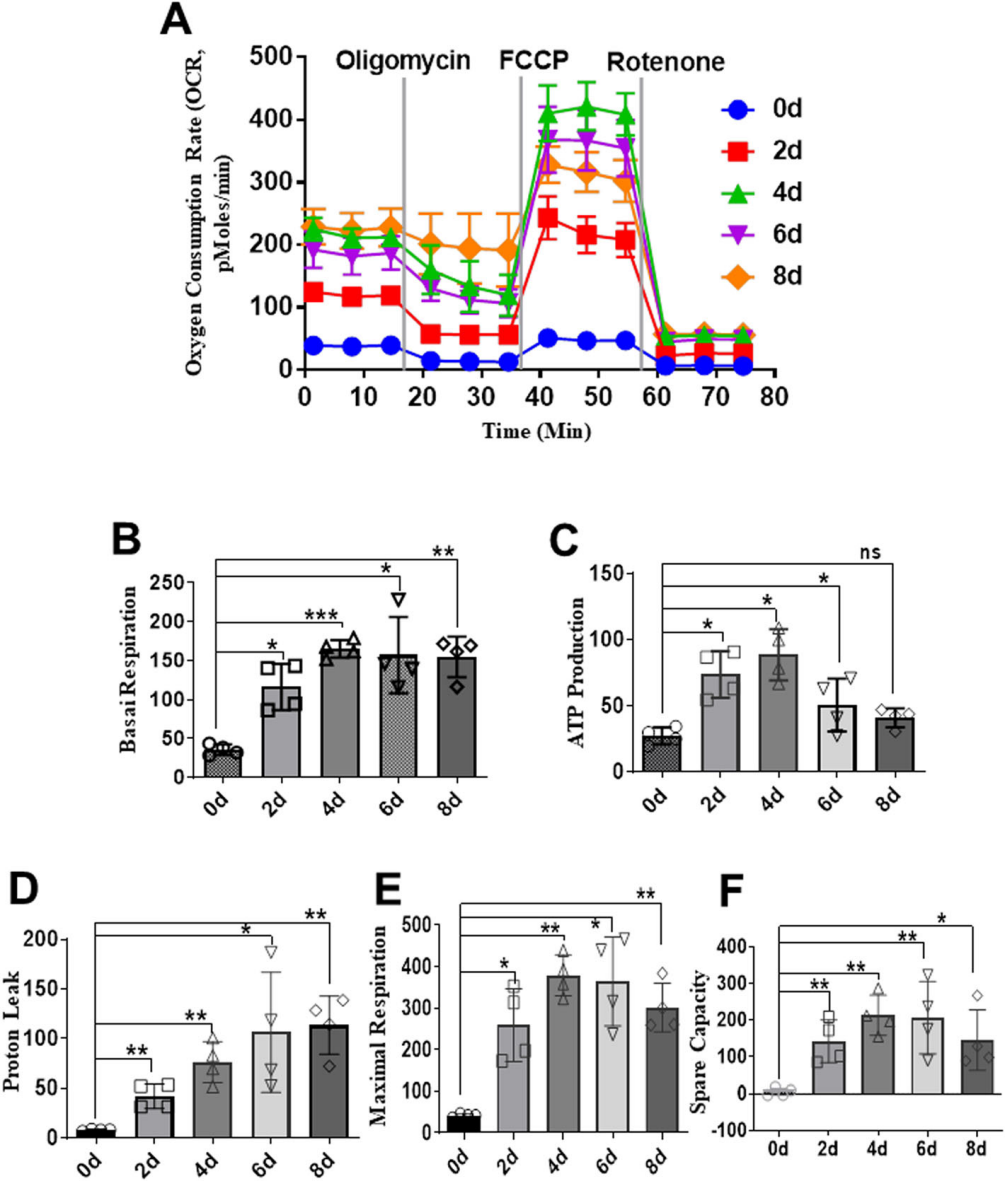
Increased mitochondrial oxygen consumption during differentiation of S-MSCs into brown adipocytes. S-MSCs were induced to differentiate for the indicated period of time (0, 2, 4, 6, or 8 days) with a brown adipogenic cocktail. **A** The oxygen consumption rate (OCR) of S-MSCs at various time points after initial stimulation was measured with an XF24 Extracellular Flux Analyzer (d) software and (e) statistically analyzed. The mitochondrial function of differentiated S-MSCs was represented by basal mitochondrial respiration (**B**), ATP production (**C**), proton leakage (**D**), maximal respiratory capacity (**E**), and spare capacity (**F**). The data are shown as the mean ± SEM of at least three independent experiments. One-way ANOVA. *P < 0.05, **P < 0.01, ***P < 0.001 versus the corresponding control; ns indicates not significant

Increased mitochondrial activity is a prerequisite for the differentiation of MSCs into adipocytes [17]. Using the Seahorse XFe96 Analyzer, we found that the oxygen consumption of S-MSCs was greatly increased from day 2 to day 8 of the differentiation period (Fig. 2a), indicating that differentiation of S-MSCs into BAs is a process that requires enhanced mitochondrial function and energy supply. Mitochondrial function, as indicated by ATP levels and mitochondrial complex activities such as basal mitochondrial respiration, proton leakage, maximal respiratory capacity, and spare capacity, was also significantly enhanced during the adipogenic differentiation process (Fig. 2b–f), suggesting that S-MSCs not only require higher baseline oxygen consumption but also exhibit increased activated mitochondrial function during BA differentiation.

### Mitochondrial biogenesis was increased during the differentiation of S-MSCs into BAs

**Fig. 3:**
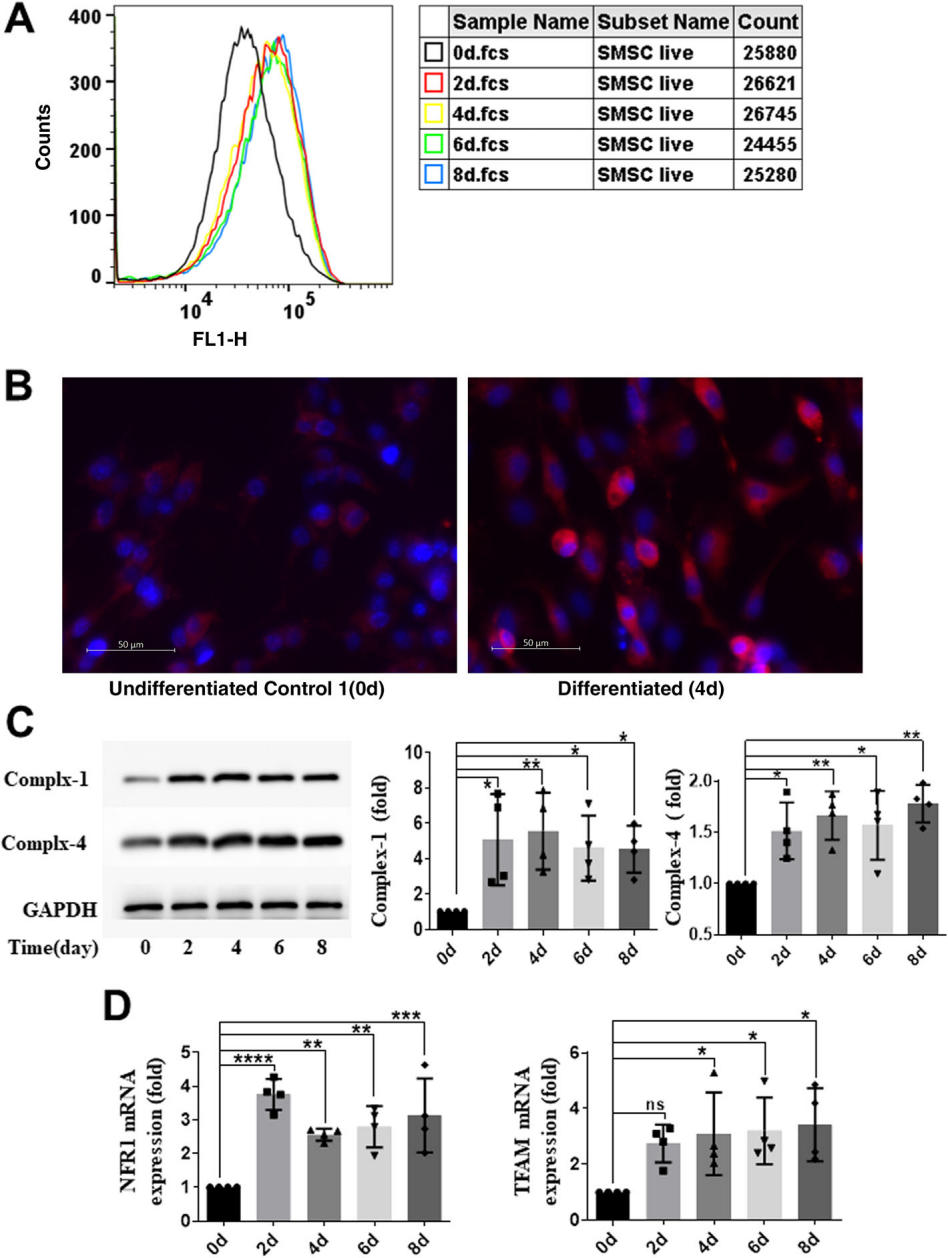
Enhanced mitochondrial biogenesis during differentiation of S-MSCs into brown adipocytes. S-MSCs were induced to differentiate for the indicated period of time. **a** The cells were stained with MitoTracker Green FM (MTGFM) and analyzed by flow cytometry to measure the mitochondrial mass on the basis of the mean fluorescence intensity (MFI). **b** Undifferentiated (0 days) and differentiated (4 days) S-MSCs were stained with MitoTracker red and DAPI and visualized by fluorescence microscopy. Original magnification: × 400. **c** Mitochondrial complex-1 and complex-4 protein expression in cells was tested by WB analysis under differentiation induction for the indicated time. Densitometric scans of three WBs are quantified in the bar graphs. The results shown are representative of three independent experiments. **d** The mRNA levels of mitochondrial transcription factor A (TFAM) and nuclear respiratory factor 1 (NRF1) in cells were measured by RT-PCR under differentiation induction for the indicated time. The data are shown as the mean ± SEM of at least three independent experiments. One-way ANOVA. *P < 0.05, **P < 0.01, ***P < 0.001 versus the corresponding control; ns indicates not significant

To further examine the role of mitochondria in brown adipogenic differentiation of S-MSCs, we measured mitochondrial mass in S-MSCs stimulated with brown adipogenic cocktail for the indicated times by staining with MTGFM and subsequent analysis by flow cytometry. The results demonstrated that mitochondrial mass was significantly increased in S-MSCs after 2 days of differentiation into BAs and remained at a relatively high level throughout differentiation into BAs (Fig. 3a). Consistently, fluorescence microscopy of cells stained with MTGFM and DAPI also showed that the mitochondrial content in cells was increased in stimulated cells compared with unstimulated S-MSCs on day 4 (Fig. 3b). Immunoblot analysis showed that the protein expression of mitochondrial complex-1 and complex-4 was robustly enhanced during differentiation into BAs (Fig. 3c). Furthermore, real-time RT-PCR revealed that the mRNA expression of mitochondrial TFAM and NRF1, the key regulating factors of mitochondrial biogenesis, was significantly upregulated during the differentiation of S-MSCs of BAs (Fig. 3d). These data confirmed that mitochondrial biogenesis is boosted during the differentiation of S-MSCs into BAs.

### Conversion of interscapular brown fat tissue to white fat tissue in Ang II-induced hypertensive mice

**Fig. 4:**
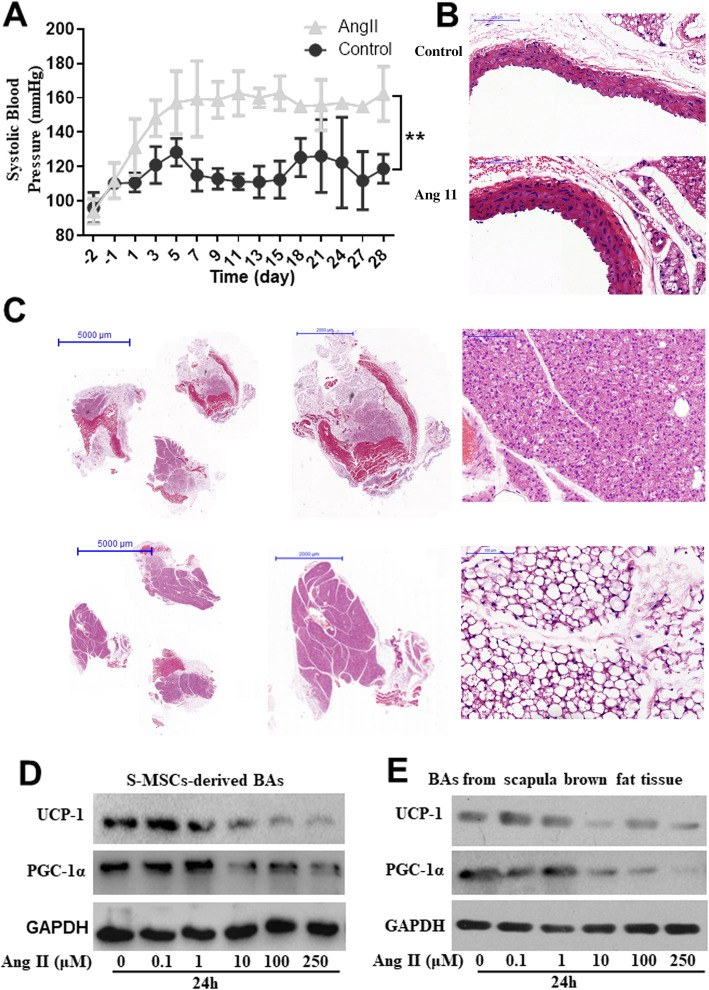
Conversion of interscapular brown adipose tissue (iBAT) to white adipose tissue (WAT) in angiotensin II (Ang II)-induced hypertensive mice. We induced hypertension in C57BL/6 (wild-type (WT)) mice (infused with Ang II at a rate of 750 ng/kg per minute for 28 days (AngII group) or 0.9% NaCl (control group)). **a** Noninvasive tail cuff monitoring of the systolic blood pressure (SBP) of the above 2 groups of mice was performed. Two-way ANOVA. *P < 0.05, **P < 0.01, ***P < 0.001 versus the corresponding control; ns indicates not significant. **b** Representative HE staining of aortic sections showing the aortic intima and media thickness (*n* = 4). **c** Representative HE staining of iBAT sections showing the conversion of BAT to WAT in iBAT from the above 2 groups of mice. Immunoblotting analysis of UCP-1 and PGC-1α protein expression in Ang II-stimulated brown adipocytes either differentiated from S-MSCs (**d**) or isolated from the iBAT of mice (**e**)

BAT is mainly composed of BAs. Animal studies have shown that transgenic ablation of BAT is associated with systemic hypertension [3]. However, the conversion of BAT in Ang II-induced hypertensive mice has rarely been investigated. Here, we employed an Ang II-induced hypertensive mouse model. During Ang II infusion at a rate of 750 ng/kg/min by Alzet osmotic pumps for 28 days, the Ang II-infused mice showed a significant increase in systolic blood pressure (SBP) and marked vascular injury, unlike control mice (Fig. 4a, b). Subsequent histological analysis revealed extensive brown-to-white adipose transformation in iBAT extracted from the Ang II-infused mice but not the control mice (Fig. 4c). For further in vitro assessment, we used Ang II to stimulate BAs either differentiated from S-MSCs or isolated from the iBATs of mice and found that the expression of the brown fat-specific marker UCP-1 and adipogenic marker PGC-1α was decreased in a dose-dependent manner after Ang II stimulation (Fig. 4d, e). Together, these data suggested that Ang II causes the whitening of BAT in hypertensive mice and induces BA dysfunction in vitro.

### Brown adipose tissue deficiency enhanced Ang II-induced hypertension and vascular remodeling

**Fig. 5:**
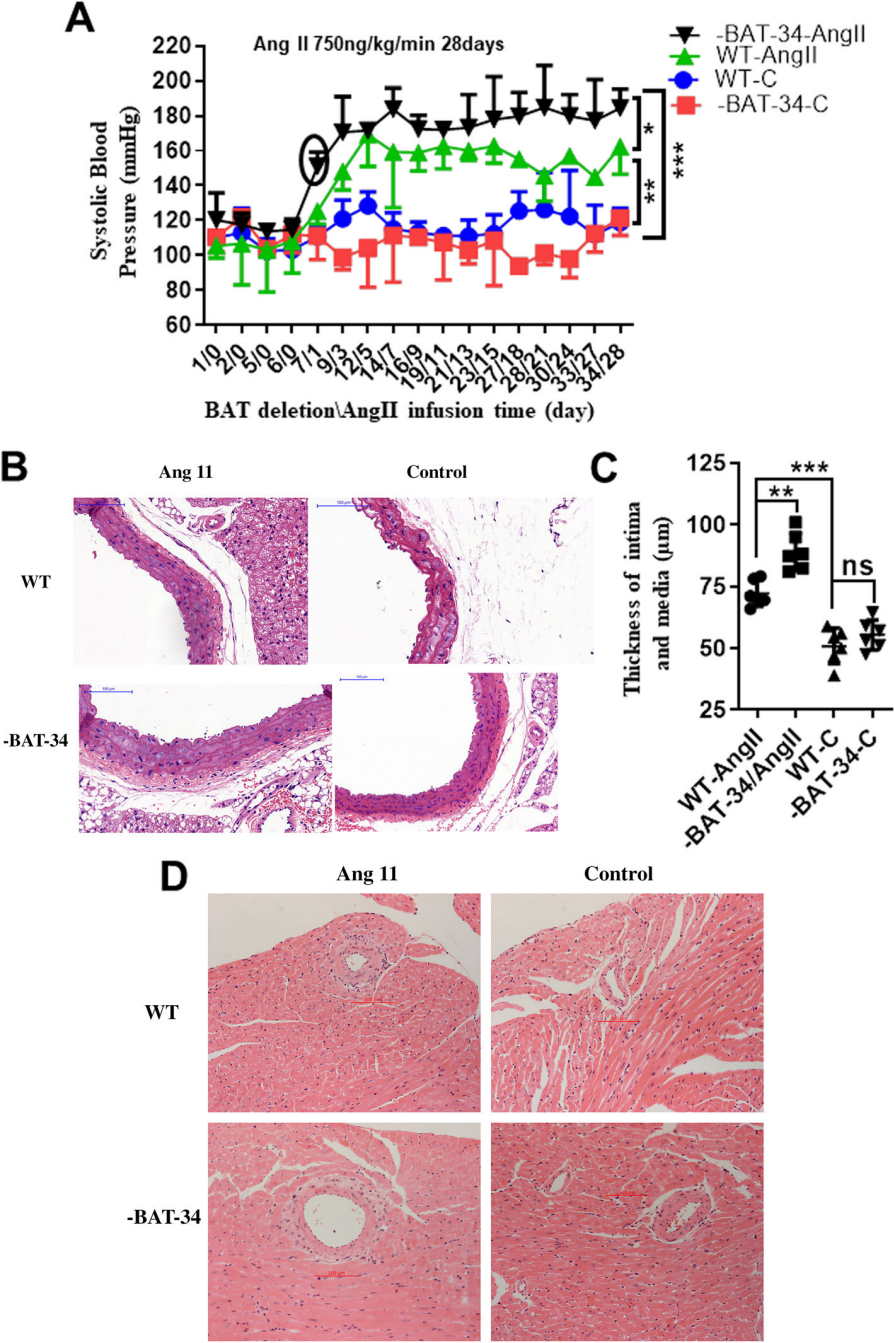
Brown adipose tissue deficiency enhanced angiotensin II (Ang II)-induced hypertension and target organ remodeling. We induced hypertension (infused with Ang II at a rate of 750 ng/kg per minute or 0.9% NaCl for 28 days) in C57BL/6 mice and established 4 groups: the WT-C group (infused with 0.9% NaCl), -BAT-34-C group (iBAT-deficient mice infused with 0.9% NaCl), WT-Ang II group (infused with Ang II), and -BAT-34-Ang II group (iBAT-deficient mice infused with Ang II). **a** Noninvasive tail cuff monitoring of the systolic blood pressure (SBP) of the above 4 groups of mice (*n* = 5–6) was performed. Student’s *t* test. **b**, **c** Representative HE staining of aortic sections and quantification of the aortic intima and media thickness (*n* = 4). One-way ANOVA. **d** Representative HE staining of heart sections (fibrotic tissues are indicated by arrows) (*n* = 4). All data that were analyzed by Student’s *t* test or one-way ANOVA passed the normality test. *P < 0.05, **P < 0.01, ***P < 0.001 versus the corresponding control; ns indicates not significant

Prompted by these results, we next aimed to determine the direct role of BAT in blood pressure (BP) regulation by using iBAT-deficient (-BAT) mice (generated by surgical removal of the iBAT depot in C57BL/6 mice, after which the animals were allowed to recover for 6 days) and WT mice. On day 7, -BAT and WT mice received Ang II or 0.9% NaCl infusion at a rate of 750 ng/kg per minute for 28 days, and BP was monitored using the noninvasive tail cuff method. We found that SBP was drastically higher in iBAT-deficient mice than in WT mice (Fig. 5a). Histological analysis of aortic sections was used to assess aortic structural changes, and HE staining showed that 4 weeks of Ang II infusion caused hypertrophy of the aorta (intima and media), which was aggravated in iBAT-deficient mice (Fig. 5b). The aortic intima and media thickness was quantified (Fig. 5c). Ang II-induced fibrosis of the heart was significantly aggravated in iBAT-deficient mice compared with WT mice (Fig. 5d). Collectively, these data suggested that deficiency of iBAT facilitates Ang II-induced BP elevation and target organ damage.

## Discussion

The ability of S-MSCs to differentiate into BAs and the molecular mechanism and role S-MSC-derived BAs have not yet been demonstrated. It is well documented that BAs are derived from distinct developmental sources, such as PDGFRα^+^ ASCs located in WAT that express the common stem cell marker Sca1, which have been shown to differentiate into BAs in vitro and in vivo [10]. BAs play a key role in the endocrine-mediated protection against hypertensive cardiac remodeling by activating the A2AR/FGF21 pathway [5]. In the present study, we revealed that S-MSCs isolated from the dermis of mouse skin were positive for PDGFRα and readily differentiated into BAs via a process regulated by mitochondrial activity in vitro*.* S-MSCs could be potential BA progenitors. Functionally, Ang II caused iBAT to be converted to WAT in Ang II-induced hypertensive mice and decreased the expression of the thermogenic functional genes UCP-1 and PGC-1α in vitro. Deficiency of iBAT markedly facilitated Ang II-induced BP elevation and target organ damage.

Recent studies have revealed that BAs can be derived from Myf5^+^ progenitor cells located in the perirenal and interscapular regions [21] and Sca-1^+^ adipogenic progenitor cells residing in murine skeletal muscle and subcutaneous white fat after bone morphogenetic protein 7 (BMP7) stimulation [22]. BAs that emerge in white fat in response to β3-adrenergic stimulation of abdominal WAT arise from the proliferation and differentiation of PDGFRα^+^ ASCs [10]. Human foetal mesenchymal stem cells have been proven to differentiate into BAs [9]. A previous study confirmed that S-MSCs can migrate to inflamed tissues and inhibit the development of atherosclerosis, EAE, and hypertension in mice and may be promising cell sources for stem cell-based therapies for chronic inflammatory diseases [12, 13, 15]. To our knowledge, no prior study has assessed the ability of S-MSCs located in the dermis to differentiate into BAs. The present study is the first to demonstrate that S-MSCs are able to readily differentiate into BAs in vitro. S-MSCs could be novel BA progenitors, similar to PDGFRα^+^ ASCs.

Mitochondria are multifaceted organelles that regulate stem cell fate decisions [20]. Generally, the activity of mitochondria in MSCs is maintained at a low level, and after MSC induction, the mitochondrial DNA (mtDNA) copy number, the OCR, mitochondrial biogenesis-related gene (NRF1 and TFAM) expression, and intracellular ATP content are increased [23]. Mitochondrial elongation is critical during the differentiation of embryonic stem cells (ESCs) into cardiomyocytes and for normal cardiac development and function [24]. Robust mitochondrial activity is a prerequisite for the differentiation of hMSCs into adipocytes [17]. The zinc finger-containing protein PRDM16 initiates the differentiation of myoblasts or white preadipocytes into functional BAs [7]. Differentiated BAs augment the expression of mitochondrial function genes (NRF1 and TFAM) and ultimately UCP1 [25]. In agreement with the previous studies described in this article, we that mitochondrial oxygen consumption and the expression of thermogenic markers (UCP1 and PGC1-α) and mitochondrial biogenesis markers (NRF1 and TFAM) were increased during the differentiation of S-MSCs into BAs.

PDGFRα^+^ S-MSCs could be novel BA progenitors. BAs are the major components of BAT. Transgenic ablation of BAT is associated with cardiovascular abnormalities and systemic hypertension in UCP-DTA mice [3]. BAT has a potential endocrine-mediated effect against hypertensive cardiac remodeling in DOCA salt-induced hypertension [5]. Tissue grafting of converted BAT has been proposed as a direct approach to increase endogenous brown fat in vivo [26]. To directly confirm the role of BAT in Ang II-induced hypertension in mice, we first assessed and compared the changes in iBAT in hypertensive mice and WT control mice. The results showed that Ang II-induced hypertension caused BAT to be converted into WAT in iBAT extracted from Ang II-infused mice but not in control mice. In vitro, we also found that Ang II stimulation caused decreased expression of UCP-1 and PGC-1α in BAs. Furthermore, we generated iBAT-deficient mice by surgically removing the iBAT depot in C57BL/6 mice and then infused the animals with Ang II, and the results demonstrated that Ang II-induced BP elevation and target organ damage were accelerated in iBAT-deficient mice compared to control mice. Overall, this study provides direct evidence that Ang II-induced hypertension is associated with BAT deficiency. The latest study demonstrated that transplantation of engineered human brown-like adipocytes prevents diet-induced obesity, ameliorates metabolic syndrome, and activates endogenous brown fat in mice [27]. This novel study provides a potential strategy to combat metabolic syndrome by using engineered human brown-like adipocytes combined with cell transfer-based therapy and provides important inspiration. The direct therapeutic effect of S-MSC-derived BAT on hypertension warrants future investigation.

**Fig. 6:**
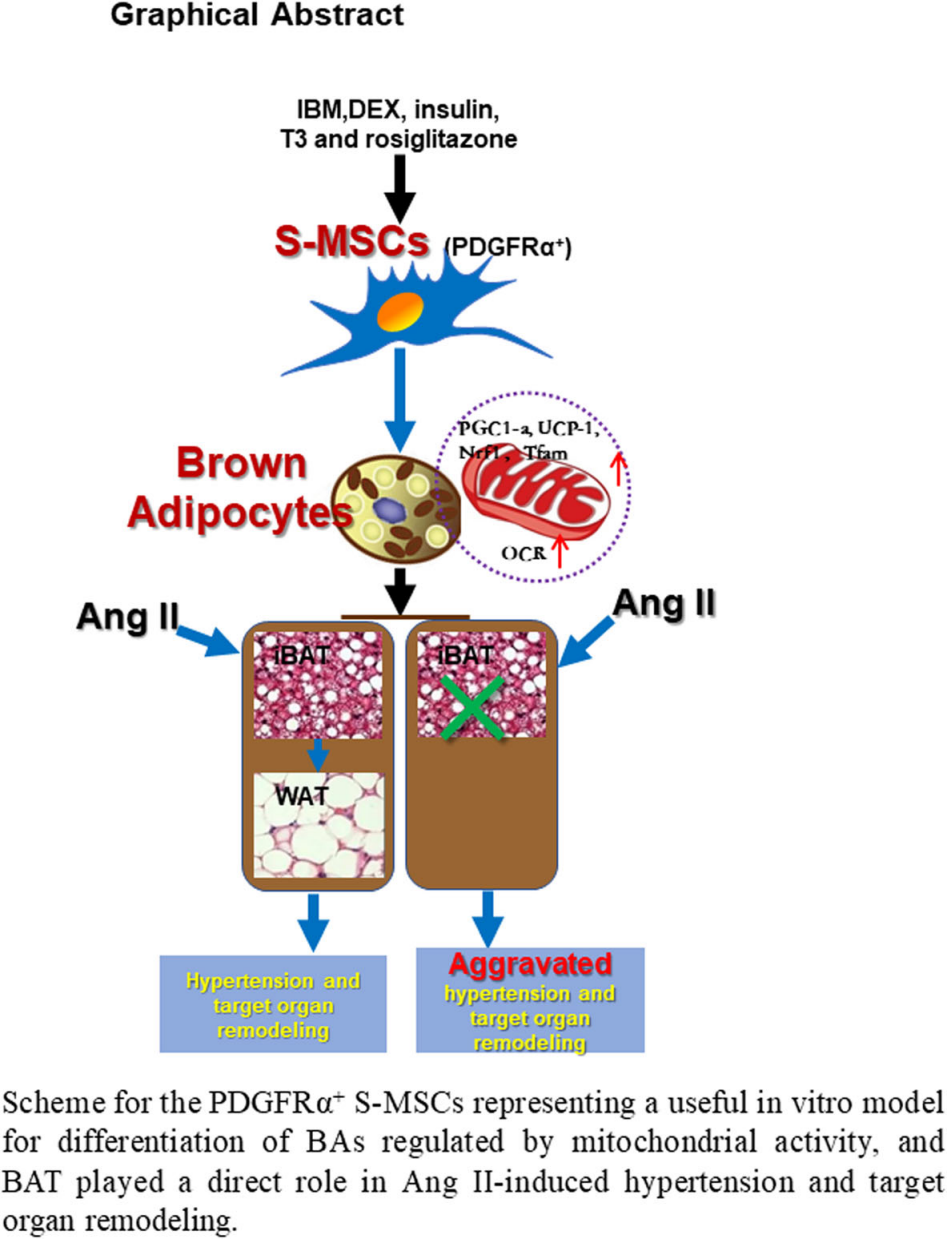
Graphical abstract. Schematic of the in vitro differentiation of PDGFRα^+^ S-MSCs into BAs via a process regulated by mitochondrial activity. BAT plays a direct role in Ang II-induced hypertension and target organ remodeling

In conclusion, we provide direct evidence that S-MSCs have the potential to easily differentiate into BAs via a process that is regulated by mitochondrial activity. Functionally, our in vitro and in vivo experiments illustrate that Ang II causes the whitening of iBAT in hypertensive mice and the dysfunction of BAs. Deficiency of iBAT markedly facilitates Ang II–induced BP elevation and target organ damage (Fig. 6). Our results suggest that S-MSCs can readily differentiate into BAs and may be potential therapeutic cell sources for the treatment of hypertension.

## Data Availability

Not applicable.

## Abbreviations

S-MSCs: Skin-derived mesenchymal stem cells
BAs: Brown adipocytes
BAT: Brown adipose tissue
ASCs: Adipose-derived stem cells
iBAT: Interscapular brown adipose tissue
WAT: White adipose tissue
OCR: Oxygen consumption rate
Ang II: Angiotensin II
FGF21: Fibroblast growth factor 21
RT-PCR: Real-time quantitative PCR
MTGFM: MitoTracker Green FM
MFI: Mean fluorescence intensity
UCP-1: Uncoupling protein 1
PGC-1α: PPARG coactivator 1 alpha
NRF1: Nuclear respiratory factor 1
TFAM: Transcription factor A
