# Correction to: Mitochondrial activity regulates the differentiation of skin-derived mesenchymal stem cells into brown adipocytes to contribute to hypertension

**DOI:** 10.1186/s13287-021-02408-4

**Published:** 2021-06-17

**Authors:** Wenda Xi, Wendong Chen, Weihong Sun, Xiangxiao Li, Zhimin Suo, Gonghao Jiang, Pingjin Gao, Qun Li

**Affiliations:** 1 The Department of Cardiovascular Medicine, State Key Laboratory of Medical Genomics, Shanghai Key Laboratory of Hypertension, Ruijin Hospital, Shanghai Institute of Hypertension, Shanghai Jiao Tong University School of Medicine, No. 197, Ruijin 2nd Road, Shanghai, 200025 China; 2 Department of Digestion, Huaihe Hospital of Henan University, Kaifeng, 475000 China

**Correction to: Stem Cell Res Ther 12, 167 (2021)**

**https://doi.org/10.1186/s13287-021-02169-0**

In the version of this paper originally published online [1], the rightmost magnified images position of the Fig. 4C was reversed, and the label of Fig. 4C was missing due to errors in typesetting. These errors have now been corrected in Fig. 4C in the original article.

The authors would also like to amend the availability of data and materials.

**Availability of data and materials section**

All data generated or analysed during this study are included in this published article.
